# Liquid Metal Patterned Stretchable and Soft Capacitive Sensor with Enhanced Dielectric Property Enabled by Graphite Nanofiber Fillers

**DOI:** 10.3390/polym14040710

**Published:** 2022-02-12

**Authors:** Priyanuj Bhuyan, Dongkyun Cho, Minjae Choe, Sangmin Lee, Sungjune Park

**Affiliations:** 1 Department of Nano Convergence Engineering, Jeonbuk National University, Jeonju 54896, Korea; priyanuj.bhuyan@jbnu.ac.kr (P.B.); xwcerl8559@jbnu.ac.kr (D.C.); chzhgksk6@jbnu.ac.kr (M.C.); rptdpa0503@jbnu.ac.kr (S.L.); 2 Department of Polymer-Nano Science and Technology, Jeonbuk National University, Jeonju 54896, Korea

**Keywords:** dielectric elastomer, graphite nanofiber, liquid metal electrode, stencil printing, stretchable and soft electronics

## Abstract

In this work, we introduce liquid metal patterned stretchable and soft capacitive sensor with enhanced dielectric properties enabled by graphite nanofiber (GNF) fillers dispersed in polydimethylsiloxane (PDMS) substrate. We oxidized gallium-based liquid metal that exhibited excellent wetting behavior on the surface of the composites to enable patterning of the electrodes by a facile stencil printing. The fluidic behavior of the liquid metal electrode and modulated dielectric properties of the composite (*k* = 6.41 ± 0.092@6 wt % at 1 kHz) was utilized to fabricate stretchable and soft capacitive sensor with ability to distinguish various hand motions.

## 1. Introduction

Dielectric elastomers classified as electroactive polymers can convert electrical energy to mechanical energy in response to strain. They are typically fabricated by dispersing conductive fillers in an insulating polymer matrix in order to enhance the dielectric properties of the polymer [1,2,3,4]. Commonly used fillers which have shown promising results include various carbon materials such as graphite [5], carbon black [6,7], carbon nanotube [8,9], graphene [10,11,12], and also other conductive entities such as liquid metal micro- and nanospheres [13,14,15], and metallic micro- and nanoparticles [16,17,18,19]. The dielectric behavior of the composites is highly governed by factors such as concentration of incorporated fillers, their structural morphology and orientation, dispersity, percolation threshold, etc. With their unique electro-mechanical properties combined with various advantages such as lightness, elastic energy, technological adaptability, thermal stability and flexibility, dielectric elastomers have found application in various fields covering skin sensors [20,21,22], actuators [21,23,24], energy harvesting [25,26,27], and artificial muscles [28,29,30].

In order to fabricate various deformable electronic devices out of these dielectric elastomers, competent electrodes need to be fabricated which can comply with the deformability of the soft dielectric layer without compensating factors such as connectivity, softness, and stretchability. Common materials used as electrodes include copper tapes, silver pastes, carbon pastes, and elastomers with highly loaded conductive fillers which are limited by their deformability or prolonged fabrication process. In lieu of them, gallium based liquid metal alloys can be utilized as soft and stretchable electrodes. Gallium and its alloys have drawn tremendous attention in the field of stretchable electronics due to their metallic conductivity, fluidity, and reconfigurability [31,32,33,34,35]. Previously, the liquid metals have been rheologically modified to make conductive pastes to serve various purposes such as conductive inks for printed stretchable circuit boards and soft 3D conductive structures [36,37,38].

In this work, by utilizing the unique features of the liquid metal electrode and the dielectric elastomer, we fabricated a soft and stretchable capacitive sensor. Polydimethylsiloxane (PDMS) has been widely used to fabricate dielectric elastomers by adding fillers such as graphene oxide [39], functionalized graphite oxide [40], carbon black [41,42], copper calcium titanate [43], barium titanate [44], and titanium di-oxide [45]. We also utilized PDMS to prepare dielectric elastomers with enhanced dielectric properties by dispersing graphite nanofiber (GNF) in the PDMS matrix. The GNFs have extensively been used in various fields such as fuel cell [46,47], selective absorption, diffusion [48,49,50,51,52,53], energy storage [54,55,56], and catalysis [57,58,59]. Here, we aimed at incorporating these conductive fillers into PDMS matrix to enhance the dielectric properties and simultaneously to preserve soft and stretchable nature of the elastomeric substrates because the GNFs are relatively soft and slippery due to crystalline structure. Gallium-based alloy EGaIn (eutectic alloy of gallium and indium) in its highly oxidized form exhibited excellent wetting behavior on the surface of the composites which enabled patterning of the electrodes by non-lithographic forced wetting method through stencil printing. The fluidic behavior of the liquid metal electrode and softness of the dielectric layer with modulated dielectric properties were harnessed to fabricate stretchable and soft capacitive sensor with ability to distinguish various hand motions.

## 2. Materials and Methods

### 2.1. Materials

Graphite nanofibers (GNF-100, diameter ~200 nm, length ~30 μm, purity >95 wt %) used as fillers were obtained from Carbon Nano-material Technology Co., LTD., Pohang, Korea. The matrix polymer used is polydimethylsiloxane (PDMS, Sylgard-184, Dow Corning). Chloroform used to disperse GNF and dilute PDMS was purchased from Samchun Chemicals, Seoul, Korea. Eutectic Gallium Indium alloy (EGaIn, 75.5% Ga, 24.5% In by weight) used for electrode preparation was purchased from Indium Corporation, Clinton, New York, USA. All materials used in this work were used without any further refinement.

### 2.2. Preparation of PDMS-Graphite Nanofiber Composites (PGNC)

**Figure 1:**
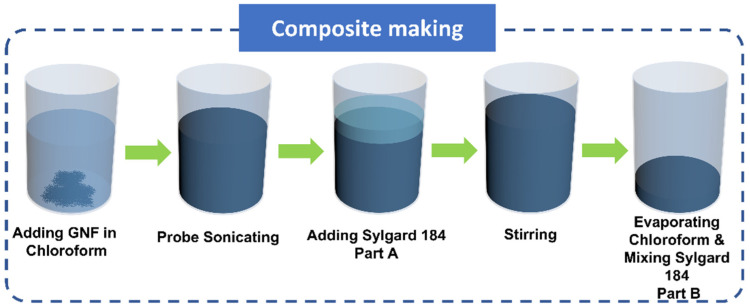
Schematic diagram of the preparation of PDMS-graphite nanofiber composite (PGNC).

Various solvent-based ultrasonication techniques to prepare homogenously dispersed composites have been reported previously [60,61,62,63]. Figure 1 shows the schematic diagram of the preparation of PGNC. The preliminary step involves dispersion of GNF in a solvent using a probe sonicator, to which subsequently PDMS pre-polymer (part A) was added followed by moderate stirring (~300 rpm) at 90 °C till complete evaporation of chloroform was attained. After that, the mixture was cooled to room temperature and curing agent (Part B, 1:10 ratio of Part A) was homogeneously mixed followed by degassing in a desiccator to eliminate air bubbles generated during mixing. Chloroform was used as the solvent as previous studies had reported both proper dispersion of carbon materials and good solubility of PDMS in it [64,65,66]. The concentration of GNF added in PDMS was in the range of 0.5–12 wt %. 

### 2.3. Preparation of Oxidized EGaIn

**Figure 2:**
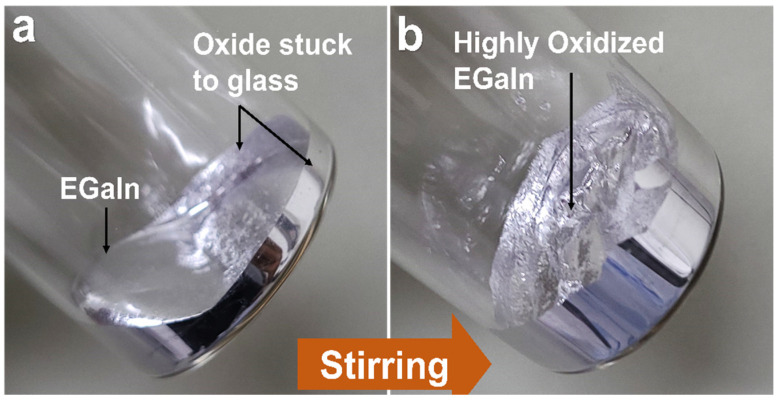
Digital pictures of a vial with EGaIn (**a**) before and (**b**) after bulk oxidation by magnetic stirring, respectively.

Oxidized EGaIn to be used as electrodes was prepared by stirring 20 g of EGaIn in a glass vial using magnetic stirrer at 200 rpm till the viscosity attained retarded the rotation of the stirrer (around 50 min) [37]. Figure 2 shows the vial with EGaIn (a) before and (b) after bulk oxidization, respectively.

### 2.4. Fabrication of Device

**Figure 3:**
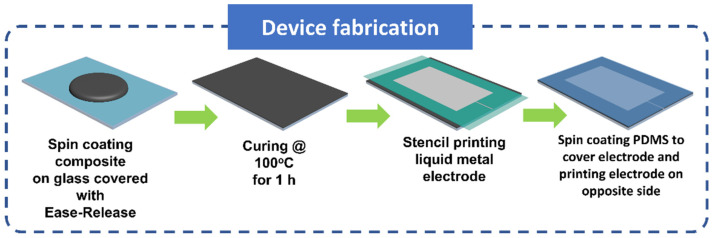
Schematic diagram of the fabrication of liquid metal patterned capacitive sensor with PGNC as dielectric layer.

Figure 3 shows the schematic diagram of fabrication of the liquid metal patterned capacitive sensor with PGNC as dielectric layer. To obtain a uniform dielectric film, the mixture as prepared in Section 2.2 was spin coated on a microscopic slide glass which had been spray coated with a releasing agent (Ease Release 200, Smooth-on). After curing the spin coated mixture at 100 °C for 1 h, the liquid metal electrode was patterned on its surface by an oxidized EGaIn coated paint brush through an OHP film stencil (cut with Cameo 4, Silhouette America Inc., Lindon, UT, USA). Next, the electrode was covered by spin coating the freshly prepared PDMS mixture on it followed by thermal curing. Similarly, the electrode was prepared on the opposite side of PDMS after peeling it off from the glass substrate to fabricate a parallel plate capacitor and thin electrical wires were sealed to the electrodes with PDMS to establish a connection with an external source.

### 2.5. Characterization

All capacitance related values were measured at room temperature using GWINSTEK 6200A precision LCR meter (Good Will Instrument Co., Ltd., New Taipei City, Taiwan). For all the dielectric related measurements, five values were recorded at each frequency points and their average values and standard errors were plotted. The thickness of the films was measured using a digital thickness gauge. Scanning electron microscopy images and optical microscopy images were obtained using Supra 40 VP (Carl Zeiss AG, Oberkochen, Germany) and CX23 (Olympus Corporation, Tokyo, Japan), respectively. All contact angle measurements were carried out using goniometer SEO-Phoenix 300 (Surface Electro Optics Co., Ltd., Suwon, Korea). DC conductivity (*σ*_dc_) of the samples were measured using KEYSIGHT 34461A benchtop multimeter (Keysight Technologies, Santa Rosa, California, USA) using the formula [67] given by Equation (1)

(1) $$\sigma_{\mathrm{dc}}=d/\;(A\times R_{p})$$

where, *d* is the thickness of the sample, *A* is the surface area, and *R_p_* is the resistance across the thickness.

## 3. Results and Discussion

### 3.1. Composite Preparation

**Figure 4:**
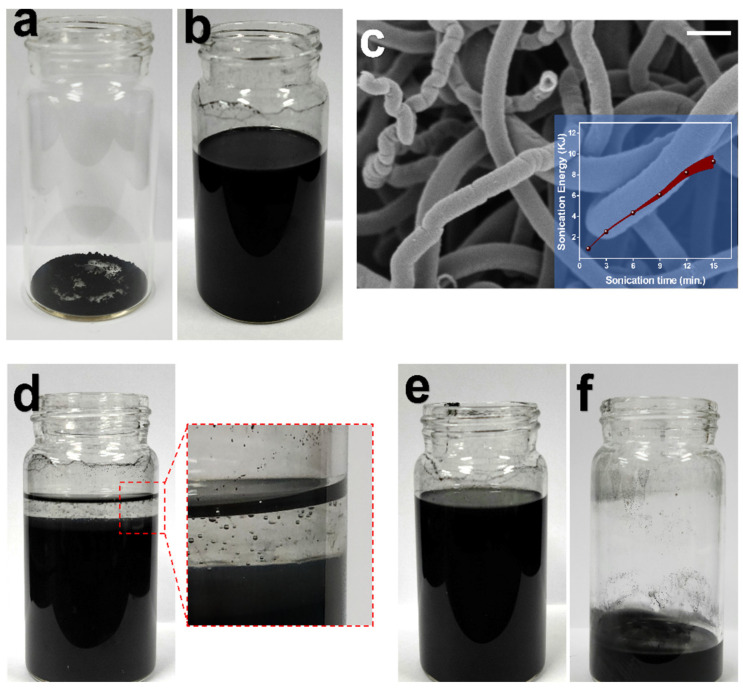
Digital images of (**a**) Graphite nanofiber (GNF) in a vial, (**b**) GNF after probe sonication in chloroform and (**c**) FESEM image of GNF dispersed in chloroform (solvent) after sonication (Scale bar represents 400 nm). Inset image shows input sonication energy in the solvent with respect to sonication duration. Digital images of (**d**) Sylgard 184-part A added to the sonicated mixture (Enlarged figure shows part A floating on the top due to density difference), (**e**) Sylgard 184-part A getting mixed during stirring and (**f**) Sylgard 184-part A mixed with GNF (part B to be added subsequently) after evaporation of solvent.

Figure 4a,b shows the images of GNF in a vial before and after sonication in chloroform. After probe sonication treatment, the solvent appeared black ensuing dispersion of GNF. Ultrasonic waves provided the required shear forces to open the entangled GNF bundles, which was confirmed by FESEM image as shown in Figure 4c and the inset figure shows the energy input in the solvent by the ultrasonic probe with respect to sonication time. After dispersion of GNF in chloroform, PDMS prepolymer (Part A) was added to the mixture and was magnetically stirred at 300 rpm on a hot plate at 90 °C Before mixing, the prepolymer could be seen floating atop the solvent mixture due to difference in density (Figure 4d). The agitation provided uniform dispersion of heat energy to slowly evaporate the chloroform and also enabled the GNF to be homogeneously dispersed in PDMS polymeric matrix resulting in GNF/PDMS Part A mixture (Figure 4e,f).

### 3.2. Dielectrc Layer

**Figure 5:**
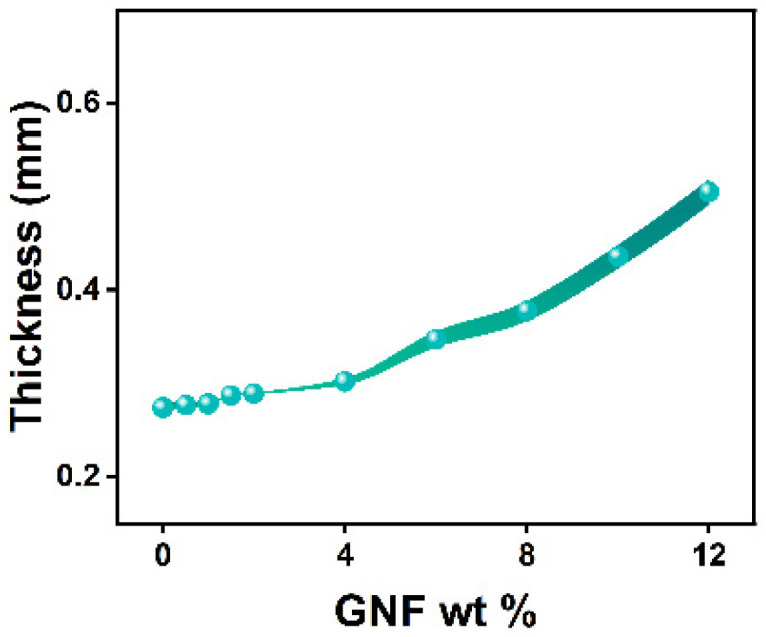
Thickness of PGNC as a function of the GNF concentration. Spin coating condition was fixed at 200 rpm for 60 s with a ramp rate of 5 s.

In order to study the effect of concentration of the GNF on the dielectric constant of PDMS, we prepared composites by varying the concentration of the GNF in the range of 0.5–12 wt %. Figure 5 shows the thickness of the spin coated composite films with respect to weight percentage of the GNF. We observed that the thickness of the films was nearly same till 2 wt % of the GNF whereas there was a moderate increase in thickness at 4 wt %. Beyond that point, the thickness increased appreciably due to the increased viscosity of the composite mixture with higher concentration of the added GNF. We stopped at 12 wt % as beyond this concentration the viscosity was too high to spin coat and the films produced were inhomogeneous in thickness, implying possible saturated mixing conditions.

### 3.3. Dielectric Properties Measurement 

The polarizability of a dielectric material is governed by its relative permittivity (*ε_r_*) or dielectric constant (*k*) which is generally expressed as a complex value [68] given by Equation (2)

(2) $$\varepsilon_{r}\left(\omega \right)=\varepsilon_{r}{}^{\prime }\left(\omega \right)-{i\varepsilon_{r}}^{\prime \prime }\left(\omega \right)$$

where the real part $\left({\varepsilon_{r}}^{\prime }\right)$ refers to the ability of the materials to interact with an external field, thereby its degree of polarization, whereas the imaginary part $\left({\varepsilon_{r}}^{\prime \prime }\right)$ indicates energy loss or energy dissipation or dielectric attenuation. The ratio of imaginary part to real part is known as the dissipation factor (*D*) which is given by the formula in Equation (3)

(3) D=tan δ

where *δ* is mathematically defined as the angle between the voltage and charging current, also known as loss angle [69]. Theoretically, at low frequencies, polarization matches with the changing electric field with minimal dielectric loss which indicates maximum contribution to the real part of Equation (2), whereas at higher frequencies the changing electric field is too fast to occur polarization [69].

**Figure 6:**
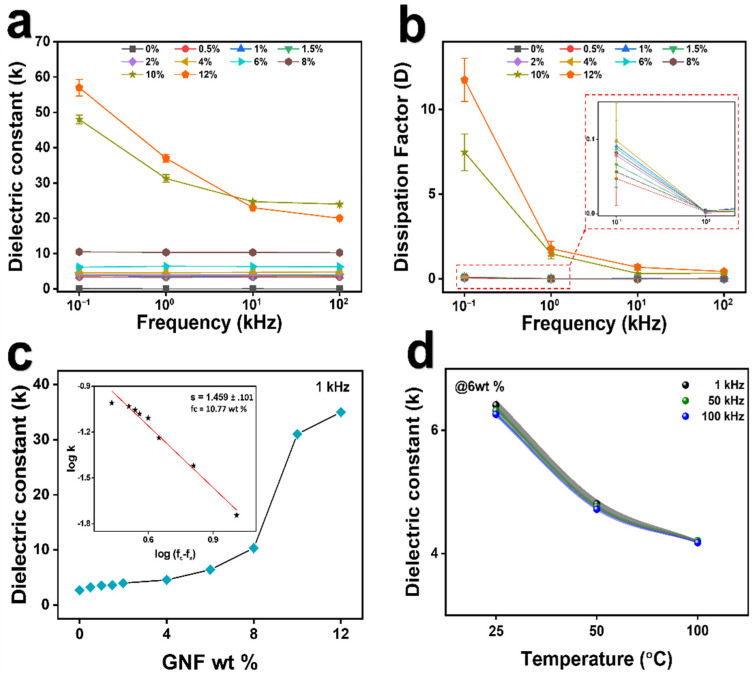
(**a**) Plot of dielectric constant (*k*) of PGNCs versus testing frequencies at different GNF wt %. (**b**) Plot of dissipation factor (*D*) of PGNCs versus testing frequencies at different GNF wt %. (**c**) Plot of dielectric constant (*k*) of PGNCs versus GNF wt % at testing frequency 1 kHz. Inset diagram shows the theoretical plot of *k* values according to Equation (4) with the highest correlation coefficient to predict the percolation threshold which was found to be ~10.77 wt %. (**d**) Plot of *k* versus temperature of PGNC with 6 wt % the GNF.

The dielectric properties of pure PDMS and PGNC with different weight percentages of the GNF measured at different frequencies are shown in Figure 6a,b. It was observed that both dielectric constant *k* and dissipation factor *D* were nearly independent of frequency up to 8 wt % of the GNF with the *D* values being very low (Figure S1). The observed steady increase in the value of *k* with the increase in concentration of the GNF in the composite films up to 8 wt % can be attributed to the interfacial polarization effect or Maxwell–Wagner–Sillars (MWS) effect originating in the insulator-conductor interface [70,71] while the values of *D* remained significantly low. At lower frequencies, the value of *k* increased sharply from 10 wt % of the GNF along with high increase in dissipation factor (Figure 6b) which is due to the transition of the composites from insulating region to semiconducting region near the percolation threshold [70] and towards higher frequencies the values of *k* and *D* both decayed owing to inability of induced charge to match the reversing field causing decrement in electronic oscillations with rising frequency [71]. Thus, it was observed that the dielectric properties were almost independent of frequency till 8 wt % of the GNF whereas above that there was sharp decrement in both *k* and *D* values with rising frequency. From this dielectric behavior, the percolation threshold can be estimated to be in the neighborhood of 10–12 wt % which was also verified by the dc conductivity values of the composites (Figure S2) which increased abruptly from 8.76E-6 S/m to 7.21E-3 S/m as the weight percentage of the GNF in PGNC increased from 8 wt % to 10 wt %. This behavior is well explained by the percolation theory which predicts the electrical properties of a composite with non-interacting randomly dispersed fillers [8]. Theoretically, the value of *k* is governed by a power law model as shown in Equation (4)

(4) $$k\;\propto {\left(f_{c}-f_{x}\right)}^{-s}$$

where *f_x_* is the weight fraction of the GNF in PGNC, f_c_ is the percolation threshold (*f_c_* > *f_x_*) and s is critical exponent in the insulating region [8]. The values of *k* were observed to be increased with the increase in weight percent of the GNF in PGNC in accordance with Equation (4) (Figure 6c and Table S1). Theoretical calculations using Equation (4) for our experimental *k* values with the highest correlation coefficient gave the best fit of our experimental results at *f_c_* = 10.77 wt % with *s* = 1.459 ± 0.101, which is a deviation from the ideal value *s* = 1, similar to previously reported literatures [71,72,73,74]. The experimental results indicate that the dielectric constant of pure PDMS increased with the addition of the GNF with low dielectric loss and experimental and theoretical percolation threshold in the neighborhood of 10 wt %. Thus, for fabrication of stretchable sensor, we set an upper limit of 6 wt % of the GNF in PDMS with an enhanced dielectric constant of 6.41 ± 0.092% at 1 kHz, in practice, this composite does not exhibit electrical conductivity—i.e., far from the threshold—whereas the PDMS composite with 8 wt % of the GNF shows conductivity upon mechanical pressuring.

Furthermore, the effect of temperature on the dielectric constant of PGNC was also studied by subjecting PGNC@6wt % the GNF to different temperatures. As shown in Figure 6d, it was observed that the value of *k* is inversely proportional to temperature as predicted by Equation (5) involving a temperature dependent function *P(T)* [75]

(5) $$k=k_{0}/P\left(T\right)$$

where *k_0_* is the dielectric constant of fillers at *P(T)* = 1. The function *P(T)* can be represented by Arrhenius equation as shown in Equation (6)

(6) $$P\left(T\right)=\xi \;e^{-E/RT}$$

where *E* refers to the vibrational activation energy of the carbon ions and ξ is the pre-factor. Theoretical studies have stated that as temperature increases, the vibrating states of carbon ions increase, preventing their alignment according to the applied electric field causing less polarization leading to decrease in *k* value [75].

A detailed comparison of some of the previously reported dielectric constant values of PDMS by incorporating different fillers at their respective concentrations of usage is shown in Table 1. From the table, it can be observed that the dielectric constant value of our PGNC is comparable to those reported in previous literatures.

### 3.4. Oxidized EGaIn as Soft and Stretchable Electrode

**Figure 7:**
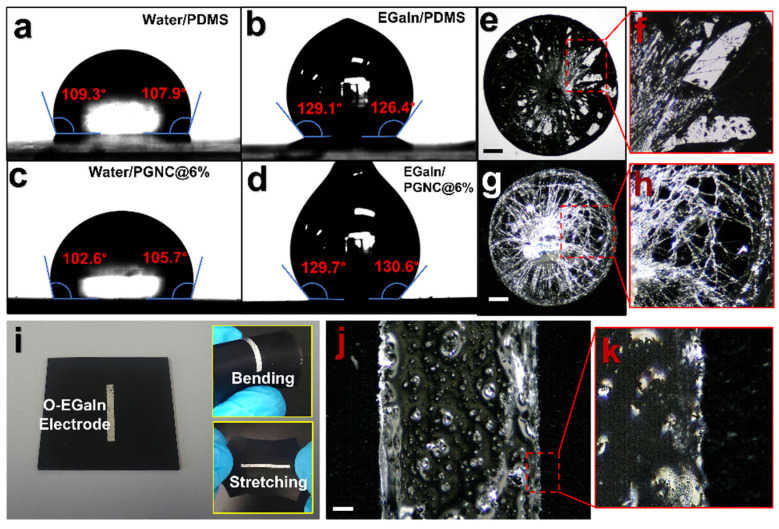
Contact angles made on PDMS by (**a**) water and (**b**) EGaIn. Contact angles made on PGNC@6wt % by (**c**) water and (**d**) EGaIn. Traces of oxide left behind on (**e**,**f**) PDMS and (**g**,**h**) PGNC@6wt % after receding EGaIn droplet (scale bars represent 400 μm). (**i**) An oxidized EGaIn electrode stencil printed on surface of PGNC@6wt %. Inset diagrams show the electrode deformation in compliance with deformation of the composite (bending and stretching). (**j**,**k**) Optical microscopy image of the electrode showing excellent wetting behavior of oxidized EGaIn on PGNC@6wt % (scale bar represents 400 μm).

Gallium based liquid metals, especially EGaIn, have attracted great interest as conducting elements for application in soft and stretchable electronics due to their remarkable properties such as “infinite” stretchability, metallic conductivity, and fluidity near room temperature [32,34,79,80,81,82,83]. In a liquid state, they can easily be injected or vacuum filled into microchannels, capillaries, and tubing under appropriate dimensional and pressure conditions or can be patterned on surface of substrates by various unorthodox non-lithograpic methods such as direct writing, stencil printing, and magnetic field guided patterning by formation of liquid metal inks [31,79,80,84]. EGaIn spontaneously forms a passivating oxide layer in air which is responsible for notoriously adhering to most substrates [85]. With an objective to fabricate soft and stretchable electrodes for the dielectric composite, we promoted oxide buildup in bulk EGaIn through magnetic stirring (Figure 2), as mentioned previously [36,37]. EGaIn without the oxide skin is a low-viscosity liquid (bulk viscosity~2 ×^−3^ Pa.s), but presence of the oxide skin imparts viscoelastic properties to the material [34]. The shear force acting on the bulk EGaIn overcomes the yield stress of the oxide layer thereby breaking it, which exposes the bulk material to air to spontaneously react with oxygen [34,86]. This breaking and reformatting the oxide layer continues till the viscosity of the material is enough to resist rotation of the stirrer ensuing formation of highly oxidized EGaIn [86] with bulk resistivity ~1.21 times that of pure EGaIn (Figure S3). This highly oxidized and viscous material could easily be deposited as electrode on the composite’s surface by stencil printing, which is a kind of forced wetting method [81,87,88]. Prior to electrode printing, the contact angles made by water and EGaIn on the composite (PGNC@6wt %) were compared to that of pure PDMS and no significant difference was observed (Figure 7a–d) which indicated negligible changes in surface wetting behaviors by the GNF fillers. Receding the EGaIn droplets left behind traces of oxide (Figure 7e–h) which signifies oxide-substrate adhesion possibly by van der Waals interaction [89]. Using the camera of the contact angle goniometer, tilting of a tiny droplet of pure EGaIn on composite was recorded (Video S1) and it was observed that the droplet adhered to the composite even when the tilt angle was 90°, signifying resistive adhesive force of the droplet’s oxide layer to the composite’s surface against gravitational force [38]. Based on our observations and previously reported literatures, we used the highly oxidized EGaIn for electrode printing due to enhanced wettability on most substrates. We used an OHP film stencil (cut with Silhouette Cameo 4) to print a small electrode on the composite (Figure 7i) which can be conformable to the applied deformations (bending and stretching) (inset of Figure 7i). Microscopic images revealed the printed area to be completely covered with the printed material with no visible traces of any unprinted spots signifying excellent wetting on the composite’s surface by oxidized EGaIn (Figure 7j,k).

**Figure 8:**
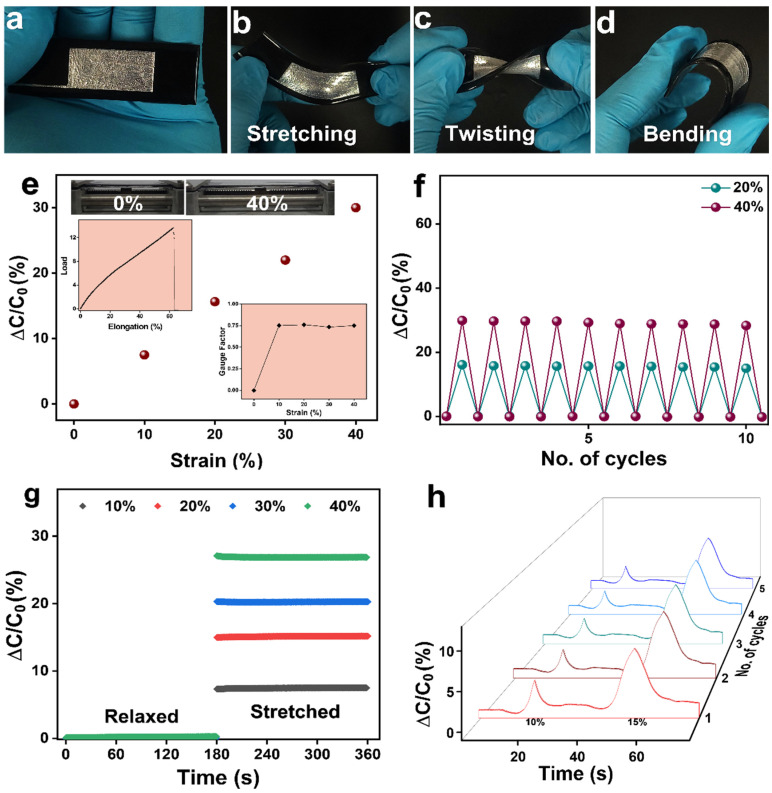
(**a**) A liquid metal electrode patterned capacitive sensor with PGNC@6 wt % as dielectric layer. The device subjected to various deformations such as (**b**) stretching (**c**) twisting and (**d**) bending. (**e**) Capacitance changes versus strain of the device. Inset images showing the load versus elongation at break (%) and gauge factor versus strain of the device. (**f**) Cyclic test of the device at different strain values. (**g**) Stability test of the device at different strain values. (**h**) Cyclic test of the device under different compression values.

Figure 8a shows a stretchable capacitive strain sensor with liquid metal electrodes (1.5 cm × 3 cm) printed on dielectric composite (PGNC@6wt %) and encapsulated by PDMS on both sides. Owing to the flexibility and stretchability of PDMS layer, dielectric layer and liquid metal electrodes, the sensor could be subjected to various degrees of deformation like stretching, twisting, and bending (Figure 8b–d). Figure 8e shows the changes in capacitance of the sensor as a function of strain. We measured the capacitance values up to 40% strain (Inset of Figure 8e). The capacitance value increased linearly as the sensor was stretched along its longitudinal axis (Figure 8e), which is due to increase in the effective area of the device and decrease in dielectric layer thickness on subjecting to strain, according to the parallel plate capacitor formula in Equation (7)

(7) $$C=k_{\;}A/d$$

where *C* is the parallel plate capacitance value, *k* is the dielectric constant of the dielectric layer, *A* is the effective area between the electrodes, and *d* is the thickness of the dielectric layer. The gauge factor of the device, which is defined as the slope of the relative capacitance change upon stretching, was found to be 0.7488 ± 0.0085 (Inset of Figure 8e). The repeatability of the capacitance value on stretching was also checked for 20% and 40% strain respectively for 10 cycles (Figure 8f) and the changes in capacitance values were observed to be uniform, demonstrating excellent repeatability of the sensor. The stability of the device at different strains was also checked by fixing the strain values (Figure 8g) and no drop in capacitance values was observed with respect to time, denoting excellent stability of the device. Similarly, the change in capacitance values of the sensor was observed for different compressive strains (Figure 8h). Under compression, the distance between the conductive fillers decreased resulting in increase in tunneling capacitance [90,91].

**Figure 9:**
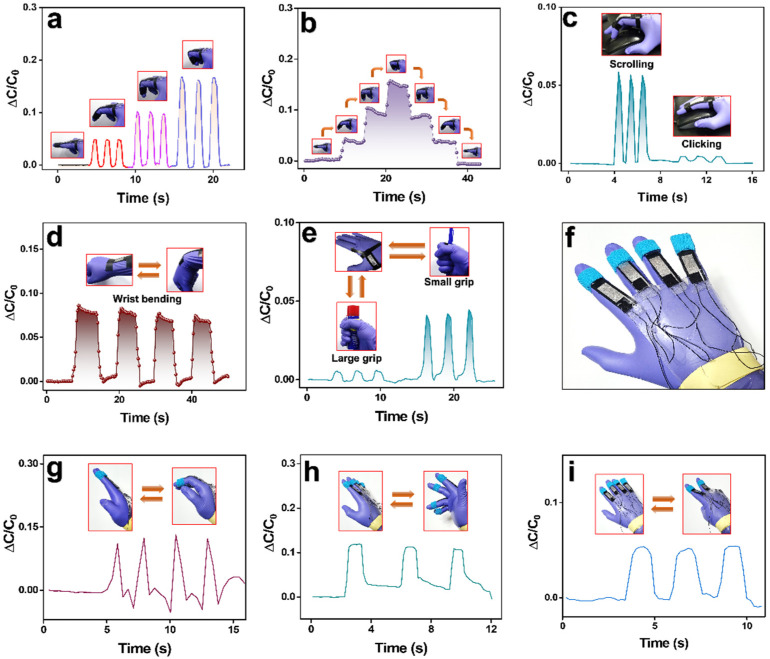
Capacitive sensor being used for various hand motions monitoring. Capacitive responses to (**a**) bending and relaxing of the index finger for various degrees of bending (**b**) step wise bending and relaxing of index finger for various degrees of bending (**c**) rolling and clicking of mouse wheel by index finger (**d**) inward wrist flexion and (**e**) pollicis brevis muscle movement while gripping objects of small and large radii. (**f**) A gesture perceiving exo-glove with capacitive strain sensors aligned along the fingers. Capacitive responses of various hand gesticulations like (**g**) calling, (**h**) finger rotating, and (**i**) victory sign.

We used our sensor to sense and distinguish between various hand motions. Figure 9a shows the capacitive change signals obtained by different degrees of bending of index finger to which the sensor has been attached. From the obtained signals, it is apparent that the higher degree of bending induces the higher deformation in the sensor, thereby resulting in increased capacitance values. Figure 9b demonstrates stepwise one cycle of bending and relaxing of the finger through various bending degrees. Each time the bending degree was fixed, the response signals were observed to attain higher and nearly fixed capacitance values. Similar responses were also recorded during stepwise relaxation of the finger to straight position which demonstrated its compliance with finger motions. This was used to monitor different work modes by the finger, such as scrolling and clicking a mouse wheel (Figure 9c). Similarly, the sensor was attached to other hand areas like wrist and pollicis brevis muscle (Figure 9d,e) and motions like inward wrist bending and gripping objects of big and small radii could be properly distinguished. Next, we conjugated four similar sensors to fabricate a smart sensing exo-glove (Figure 9f) which was used to distinguish various gesticulations such as calling, fingers rotating, and victory sign (Figure 9g–i) indicating its possible use as a gesture perceiving glove for application as a smart human motion monitoring device.

## 4. Conclusions

In this work, we introduced soft and stretchable liquid metal (EGaIn) electrode patterned capacitive strain sensor with PDMS/graphite nanofiber composite (PGNC) as dielectric material. The dielectric properties of pure PDMS were enhanced by homogeneous dispersion of graphite nanofibers as fillers in the PDMS matrix. EGaIn was highly oxidized by magnetic stirring, which showed excellent wetting characteristics on PGNC and imparted suitable rheology for stencil printing of liquid metal electrodes on either surface of PGNC. The synergistic combination of enhanced dielectric properties of the soft and stretchable composite and the characteristic stretchability and viscoelasticity of the patterned electrodes enabled fabrication of soft and stretchable capacitive sensors that were used to sense and monitor various human motions, including finger movements and hand gesticulations, owing to capacitance changes induced by different degrees of deformation of the sensor. A combination of ease in fabrication, deformability and applicability establishes the versatility of the soft and stretchable sensor which can further prove competency in conformal, wearable, and stretchable electronics and soft robotics.

## Figures and Tables

**Table 1:** Dielectric constant values of PDMS composites with various fillers.

Filler	Dielectric Constant Value	Concentration
Graphene Oxide	3.4–9.6 [39]	0.5 vol %
PDA@SiO_2_@GO ^1^	~6 [40]	6 wt %
SrTiO_3_ ^2^	~14 [76]	30 vol %
Carbon Black	~6.5 [42]	4 wt %
CCTO ^3^	6.5 [43]	20 wt %
BaTiO_3_ ^4^	~5 [44]	40 wt %
TiO_2_ ^5^	~4.5–4.9 [77]	8–10 vol %
TiO_2_@SiO_2_ ^6^	~7 [45]	16 vol %
Ag@SiO_2_ ^7^	6.8 [78]	3 wt %
GNF (this work)	6.41	6 wt %

^1^ Polydopamine modified silicon dioxide@graphite oxide hybrid. ^2^ Strontium titanate. ^3^ Copper calcium titanate. ^4^ Barium titanate. ^5^ Titanium dioxide. ^6^ Core–shell structured titanium dioxide@silicon dioxide. ^7^ Silver coated silicon dioxide nanoparticles.

## Data Availability

The data presented in this study are available on request from the corresponding author.

## Supplementary Materials

The following are available online at https://www.mdpi.com/article/10.3390/polym14040710/s1. Figure S1: Plot of dissipation factor (D) of PGNCs versus testing frequencies up to 8 wt% GNF; Figure S2: DC conductivity of PGNCs with different loadings of GNF fillers; Figure S3: Resistivity values of pure EGaIn and highly oxidized EGaIn; Table S1: Dielectric constant (k) of PGNCs at different GNF wt% at testing frequency 1 kHz. The dielectric values are measured at room temperature. Video S1: Adhesion test of a tiny droplet of EGaIn on the surface of PGNC@6 wt% by tilting.
