# Supplementary material for: Liquid Metal Patterned Stretchable and Soft Capacitive Sensor with Enhanced Dielectric Property Enabled by Graphite Nanofiber Fillers
Source: Polymers (Basel). 2022 Feb 12;14(4):710. doi: 10.3390/polym14040710 (PMC8879769; doi:10.3390/polym14040710)
Supplement: Supplementary file 1 [file polymers-14-00710-s001.zip › polymers-1571386-SI.pdf]

polymers

Supplementary Materials

Liquid Metal Patterned Stretchable and Soft Capacitive Sensor with Enhanced Dielectric Property Enabled by Graphite Nanofiber Fillers

Priyanuj Bhuyan [1,2], Dongkyun Cho [1,2], Minjae Choe [1,2], Sangmin Lee [1,2] and Sungjune Park [1,2,*]

1 Department of Nano Convergence Engineering, Jeonbuk National University, Jeonju 54896, Korea; priyanuj.bhuyan@jbnu.ac.kr (P.B.); xwcerl8559@jbnu.ac.kr (D.C.); chzhgksk6@jbnu.ac.kr (M.C.); rptdpa0503@jbnu.ac.kr (S.L.)
2 Department of Polymer-Nano Science and Technology, Jeonbuk National University, Jeonju 54896, Korea
* Correspondence: s.park@jbnu.ac.kr

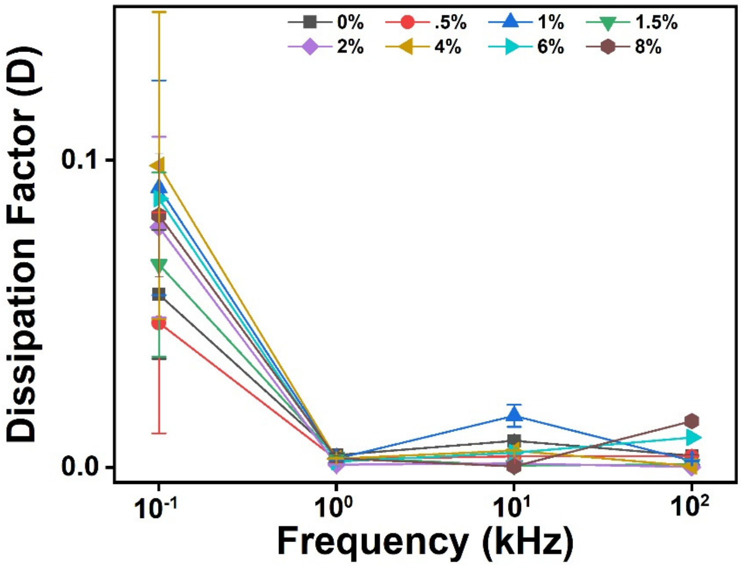

Figure S1. Plot of dissipation factor (D) of PGNCs versus testing frequencies up to 8 wt % GNF.

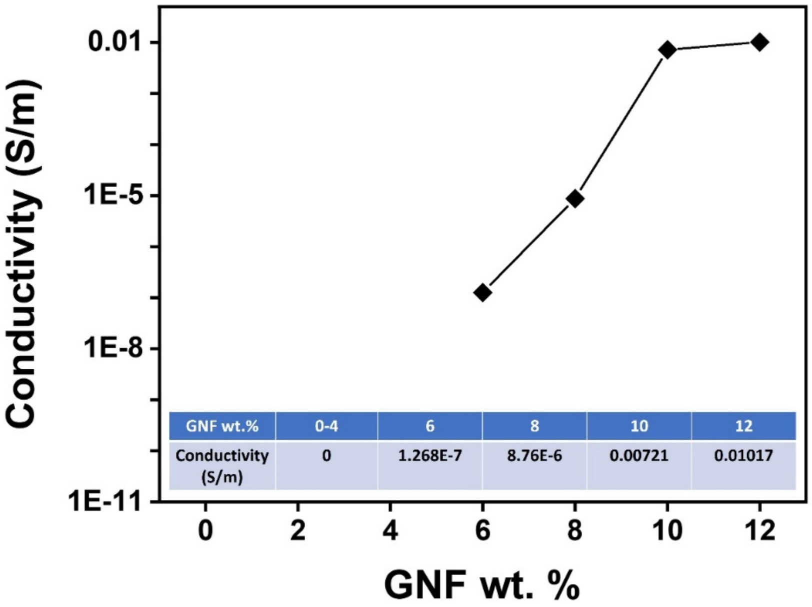

Figure S2. DC conductivity of PGNCs with different loadings of GNF fillers.

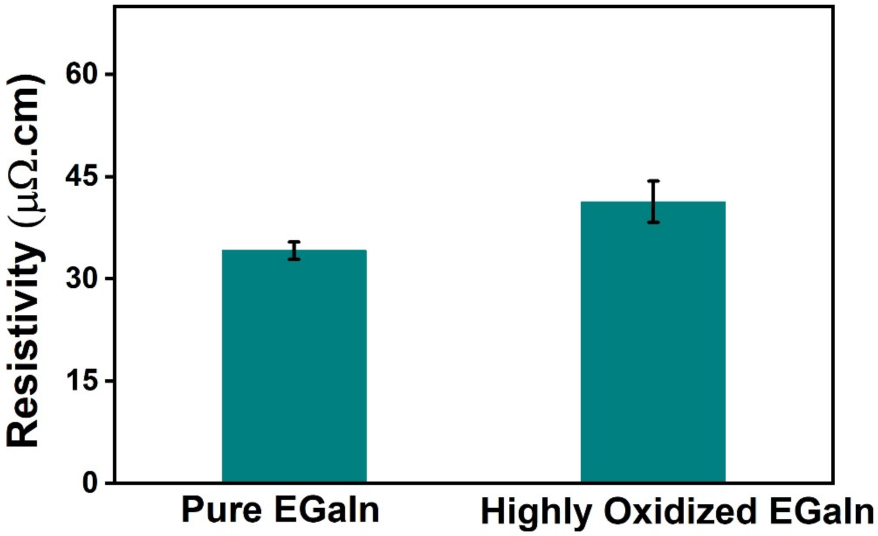

Figure S3. Resistivity values of pure EGaIn and highly oxidized EGaIn.

Table S1. Dielectric constant (k) of PGNCs at different GNF wt % at testing frequency 1 kHz. The dielectric values are measured at room temperature.

GNF wt %	Dielectric Constant
0	2.7

Copyright: © 2022 by the authors. Licensee MDPI, Basel, Switzerland. This article is an open access article distributed under the terms and conditions of the Creative Commons Attribution (CC BY) license (https://creativecommons.org/licenses/by/4.0/).

0.5	3.24
1	3.53
1.5	3.63
2	3.98
4	4.56
6	6.41
8	10.34
10	31
12	35

Supplementary Video List

Video S1. Adhesion test of a tiny droplet of EGaIn on the surface of PGNC@6 wt % by tilting.
